# Recent Changes in the Personnel of the United States Bureau of Animal Industry

**Published:** 1897-01

**Authors:** 


### CORRESPONDENCE.

Recent Changes in the Personnel of the United States Bureau of Animal Industry. The resignations of Dr. V. A. Moore, chief of the pathological division, and of Dr. Pierre Fish, first assistant, each of whom had accepted chairs in the New York State Veterinary College, at an increase of more than 35 per centum in their salaries, forms another step in the regretable series of losses of valuable trained men to the public service.

Dr. Moore, though chief of division but little over a year, has been in the laboratory over eight years, and is the author of valuable treatises on hog-cholera, swine-plague, turkey and chicken diseases, milk examination, and bacteriological technique. The New York State College is fortunate to have secured his services.

Dr. Fish had been in the laboratory a much shorter period, and had not yet come prominently before the public in a veterinary line. He was highly skilled in laboratory technique, and is the author of several important papers on the structure of the brain. His appointment to an important chair shows the high standing in which he is held as an investigator and teacher.

On November 15th last the place vacated by Dr. Moore was filled by the transfer of Field Inspector Dr. Victor A. Norguard, who has been connected with the Bureau about five years, is the author of an article on actinomycosis, and has lately been engaged in experiments in dipping cattle to remove ticks.

Dr. Charles Dawson, long an assistant in the Bureau, has been deservedly promoted to first assistant's place.

The services of Dr. Cooper Curtice, live-stock agent of the Bureau, were terminated November 30th on account of the discontinuance of temporary service inspecting cows for tuberculosis and the completion of other work to which he had been assigned.

The repeated losses sustained by the Bureau in exchanging men of long training in special branches for those comparatively unknown and of limited experience are such as to seriously interfere with its work. No sooner do incumbents become fitted for their special work and show marked adaptation and ability in prosecuting it than they are withdrawn to fill chairs in the universities, where their work is more highly appreciated. The Bureau will never be eminently successful in its investigations until it is prepared to employ and retain in its service the most skilful men of the country. The little already attained has been accomplished by men in the early years of their training, their more mature work being secured by the institutions they have joined.

Though the civil-service reform has in many ways improved the general service, it has yet another step to take in recognizing the fact that men most competent to fill certain scientific government positions obtain far higher salaries outside, and the grading of these positions with those of the purely clerical force serves to keep them filled with inexperienced men. For example, in this Bureau alone two men who have added the most fame to its scientific reputation by reason of personal work have within nearly a year left the division of pathology to take chairs at greatly increased salaries. By statutory provision they could receive as chief of division but twenty-two hundred and fifty dollars, in accordance with the theory of graded service, which demands that heads of all divisions, whether of the Treasury, Interior, or Agricultural Departments, shall receive but twenty-five hundred, or in this case less. Up to this time the theory has not been effective in retaining the best service. Congress, in appropriating four thousand dollars for the Chief of Bureau, recognizes the importance of the place. Its next step is to recognize the value of the best scientific work, pay the workers commensurately with the Chief of Bureau, and employ men whose attainments rank them among the first of the country.

VETERINARY DEPARTMENT, UNIVERSITY OF CALIFORNIA.
SAN FRANCISCO, CAL., November, 1896.

THE EDITOR JOURNAL OF COMPARATIVE MEDICINE:

DEAR SIR: At a meeting of the students of the above College, held this day, it was unanimously

*Resolved,* That this meeting having read the remarks of Dr. Archibald in reference to the above College in his report to the United States Veterinary Medical Association, published in the JOURNAL OF COMPARATIVE MEDICINE for this month, it is

*Resolved,* That this meeting tenders to each and every professor a hearty vote of thanks for the untiring efforts they have made to promote the welfare of this College and for the disinterested way in which each and every one of them has labored for the advancement and welfare of the students. It was further

*Resolved,* That the editor of the JOURNAL OF COMPARATIVE MEDICINE and the Secretary of the United States Veterinary Medical Association be informed that the statements made are false from beginning to end, and were made solely in a cowardly spirit of revenge by an individual whose efforts to obtain a position on the teaching staff were fortunately frustrated.

JOS. A. WELSH, on behalf of Class '97,
J. OTIS JACOBS, on behalf of Class '98,
THOMAS E. CARROLL, on behalf of Class '99,
Committee.

